# Prenylation-dependent membrane localization of a deubiquitinating enzyme and its role in regulating G protein–mediated signaling in yeast

**DOI:** 10.1016/j.jbc.2025.108180

**Published:** 2025-01-10

**Authors:** Fangli Weng, Xin Jin, Sindhu Ragunathan, Shan Huang, Thomas Kane, Matthew Stoeckel, Yuqi Wang

**Affiliations:** Department of Biology, Saint Louis University, St Louis, Missouri, USA

**Keywords:** prenylation, Miy1, deubiquitination, Gpa1, G protein, pheromone signaling, yeast

## Abstract

Miy1 is a highly conserved deubiquitinating enzyme in yeast with MINDY1 as its human homolog. Miy1 is known to act on K48-linked polyubiquitin chain, but its biological function is unknown. Miy1 has a putative prenylation site, suggesting it as a membrane-associated protein that may contribute to the regulation of cell signaling. Here, we demonstrate that Miy1 is localized in the plasma membrane and nuclear periphery. Mutating the putative prenylation site in Miy1 or disrupting the farnesyltransferase activity impairs its localization. Consistent with a role of Miy1 in regulating the ubiquitination status of membrane proteins, the *miy1Δ* mutants exhibit a higher level of ubiquitinated conjugates at the plasma membrane. To examine a role of Miy1 in regulating cell signaling across plasma membrane, we focused on the pheromone response, as both Ste2, the receptor for mating pheromone, and Gpa1, the cognate Gα protein of Ste2, are well known to be regulated by ubiquitination. We find that Miy1 interacts with Gpa1, regulates its level of ubiquitination and abundance. Pheromone-induced MAP kinase Fus3 activation is also altered in the *MIY1*-disrupted mutants. The findings demonstrate that Miy1 is a membrane-associated deubiquitinating enzyme and a regulator of G protein–mediated signaling.

The ubiquitin system is one of the most important regulators of cell function (1, 2, 3, 4). Ubiquitination has been shown to play a key role in the regulation of the cell cycle (5, 6), gene transcription (7, 8), and development (1, 4). Increasing evidence shows that ubiquitination also plays a key role in the regulation of G protein signaling (9, 10). In mammalian cells, several heterotrimeric G protein subunits have been demonstrated to be regulated by the ubiquitination pathway (9, 10). Well-documented examples include Gαo (11), Gαi3 (12), Gαi2 (13), Gαs (14), Gγ2 (15), and transducin (16). In yeast, ubiquitination of cell surface G protein–coupled receptors (Ste2, Ste3) functions as a signal for ligand-induced endocytosis (17, 18). The G protein α subunit Gpa1 also undergo ubiquitination, which serves to regulate both its localization and abundance (9, 19, 20).

An important factor that contributes to the versatility of ubiquitination in cell regulation is the reversible nature of the process. Removal of the conjugated ubiquitin from a substrate is catalyzed by deubiquitinating enzymes, most of which are highly conserved (21, 22). There are a total of 21 deubiquitinating enzymes in yeast and more than 100 in humans (23). The activity of these enzymes can be very specific for the substrate proteins and/or for a specific type of ubiquitin linkages. Some well-established examples include histone deubiquitination by Ubp8 (24), p53 deubiquitination by both Usp7 and Usp10 (25, 26), free K48-linked ubiquitin-chain disassembly by Ubp14 (27), and removal of K63-linked ubiquitin chain from TRAF6 by A20 (28, 29). Because of their crucial function in cell regulation and disease processes, deubiquitinating enzymes have emerged as potential drug targets, and inhibitors acting on these enzymes are pursued for therapeutic purposes (30, 31). For example, Usp7 inhibitors are tested in preclinical stage for treating multiple myeloma and chronic lymphocytic leukemia (29).

MINDY is a newly discovered family of deubiquitinating enzymes present in all eukaryotes (32). They are highly selective for K48-linked polyubiquitin chain and work by removing the ubiquitin moiety from the distal end of the polyubiquitin chain (32, 33). So far, very little is known about their biological functions (32, 34). Miy1 (also known as Miy2), encoded by the ORF YPL191C, is a yeast homolog of MINDY. Yeast also has another homolog, named Miy3 here, and is encoded by YGL082W. The sequences of all MINDY members, including Miy1 and Miy3, possess a putative prenylation site at the C-terminal end (35), suggesting that they may undergo prenylation. Prenylation could anchor these proteins to the plasma membrane, allowing them to serve as deubiquitinating enzymes to regulate the ubiquitination status of plasma membrane proteins. In this study, we tested this hypothesis in yeast and demonstrated that both Miy1 and Miy3 can indeed be found in the plasma membrane in a prenylation-dependent manner, and that Miy1 plays a role in limiting the extent of ubiquitination of plasma membrane proteins. We also provide evidence that Miy1 plays a role in pheromone signaling by regulating both the ubiquitination and abundance of Gpa1, the Gα subunit of a heterotrimeric G protein in the yeast pheromone pathway.

## Results

### Prenylation-dependent subcellular localization of Miy1 and Miy3

MINDY1 is the newest addition of deubiquitinating enzymes in mammals (32). MINDY1 is highly conserved with two homologs in yeast, which are encoded by two uncharacterized ORFs YPL191C and YGL082W, respectively. The homolog encoded by YPL191C was named Miy1 for MINDY1 in yeast in one study (32) and was renamed as Miy2 in another study by the same group (36). To avoid confusion of nomenclature, here, we name the homolog encoded by YGL082W Miy3. Our goal is to characterize the subcellular localization of Miy1 and Miy3 and to elucidate their physiological functions.

**Figure 1:**
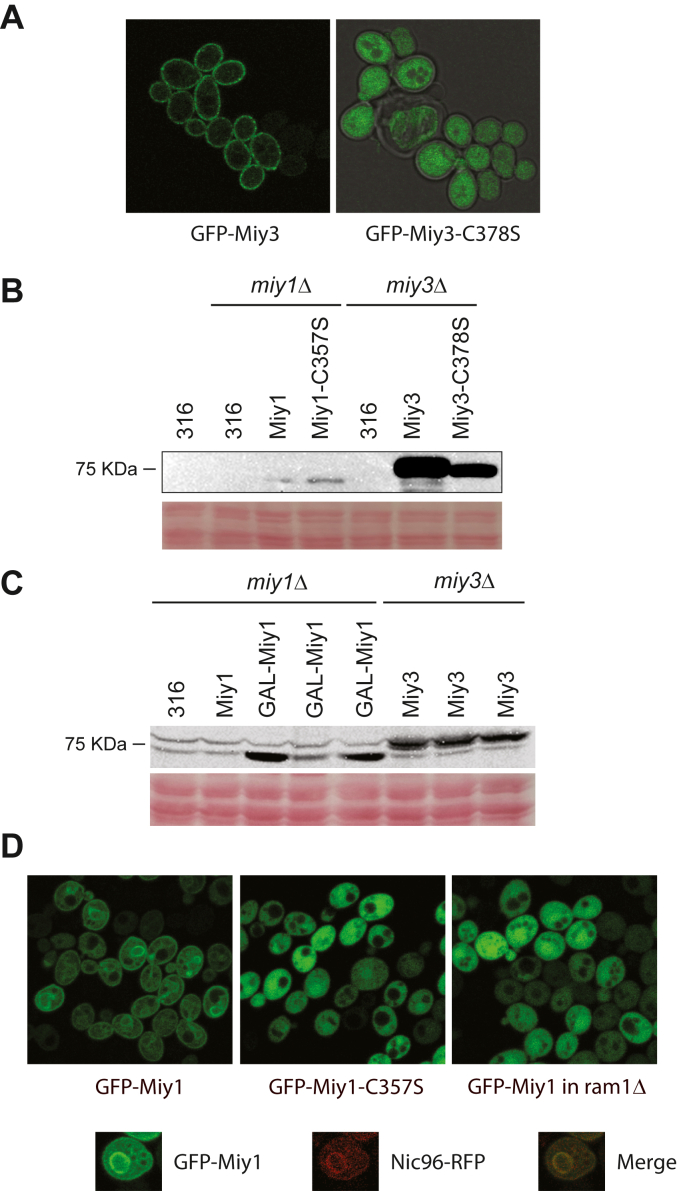
**Prenylation-dependent subcellular localization of Miy1 and Miy3.***A*, WT cells expressing either GFP-Flag-Miy3 or GFP-Flag-Miy3-C378S under the native *MIY3* promoter were grown to mid-log phase, and the localization of GFP-Flag-tagged proteins were analyzed using confocal microscopy. *B*, cells were transformed with either an empty pRS316 vector (316) or pRS316 plasmid that expresses GFP-Flag-tagged Miy1, Miy1-C357S, Miy3, or Miy3-C378S under the native *MIY1* or *MIY3* promoter and grown to mid-log phase. Whole-cell extracts were analyzed by Western blot using anti-Flag. Equal loading was confirmed by Ponceau S staining. *C*, cells were transformed with either an empty pRS316 vector (316) or pRS316 plasmid that expresses GFP-Flag-tagged Miy1 (Miy1), or Miy3 under the native *MIY1* or *MIY3* promoter, or plasmid that expresses GFP-Flag-tagged Miy1 under the control of a strong *GAL1* promoter (GAL-Miy1) and grown to mid-log phase. Whole-cell extracts were analyzed by Western blot using anti-Flag. Three individual colonies of GAL-Miy1 and Miy3 were included. Equal loading was confirmed by Ponceau S staining. *D*, WT cells expressing either GFP-Flag-Miy1 or GFP-Flag-Miy1-C357S under the control of a strong *GAL1* promoter or the *ram1Δ* mutant cells expressing GFP-Miy1 under the control of a strong *GAL1* promoter were grown to mid-log phase in galactose medium, and the localization of GFP-tagged proteins were analyzed using confocal microcopy. Colocalization with Nic96-RFP was shown in the *lower panel*.

Like MINDY1, both Miy1 and Miy3 have a putative prenylation site at the C-terminal end (CILL in MINDY1, CVVM in Miy1, and CVIM in Miy3), suggesting these proteins may be plasma membrane-associated. To test this, we fused a GFP tag to the N terminus of Miy1 and Miy3 and examined the subcellular localization of these proteins using confocal microscopy. To minimize the potential effects of overexpression on subcellular localization, both GFP-tagged proteins were expressed under the control of their native promoter and in a single-copy plasmid. Under this condition, we were able to clearly detect GFP-Miy3 signal but not GFP-Miy1 signal. As shown in Figure 1*A*, consistent with our hypothesis, GFP-Miy3 is clearly found in plasma membrane. To examine whether the putative prenylation site in Miy3 is required for its appropriate localization of Miy3, we mutated the cysteine residue, that is, Cys^378^, in its CAAX box to a serine residue and examined its localization. As shown in Figure 1*A*, distinct from the WT Miy3 protein, the Miy3^C378S^ mutant is not present in plasma membrane but in cytoplasm instead.

Our inability to detect GFP-Miy1 may be due to a lower expression level of Miy1 than Miy3. To verify this, we compared the levels of epitope-tagged Miy1 and Miy3 when both tagged proteins are expressed under the control of their native promoter and in single-copy plasmid. As shown in Figure 1*B*, the level of Miy1 is indeed much lower than that of Miy3. To facilitate our investigation of the subcellular localization of Miy1, we expressed GFP-tagged Miy1 under a strong inducible *GAL1* promoter (Fig. 1*C*). As shown in Figure 1*D*, GFP-Miy1 is primarily found in plasma membrane as well as the periphery of some intracellular organelles. To ascertain the identity of these organelles, we made the use of a variety of organelle marker proteins that are fused with red fluorescence protein (RFP) and examined the colocalization of GFP-Miy1 and those RFP-fused marker proteins. We can consistently observe the colocalization of GFP-Miy1 and Nic96-RFP (Fig. 1*D*). Nic96 is a component of nuclear pore complex, thus the colocalization of GFP-Miy1 and Nic96-RFP suggests that a portion of GFP-Miy1 is present in the nuclear envelope. Overexpression of a protein could impact its subcellular localization (37); thus, it is possible the presence of Miy1 in the nuclear envelope was a result of its overexpression. To test whether prenylation is required for appropriate subcellular localization of Miy1, we mutated its putative prenylation site Cys^357^ to a serine residue and examined its localization. As shown in Figure 1*D*, the Miy1^C357S^ mutant is no longer present in plasma membrane or the nuclear periphery. Rather, it is found in the cytoplasm. As an alternative approach to determine the requirement of prenylation on proper Miy1 localization, we expressed GFP-Miy1 in the *ram1Δ* mutant, in which Ram1, a catalytic subunit of yeast farnesyltransferase (38), is deleted. As expected, disrupting Ram1 severely impairs the proper localization of GFP-Miy1 in a manner like the prenylation site mutant GFP-Miy1^C357S^. Based on these results, we conclude that both Miy1 and Miy3 are membrane-associated and prenylation is required for their proper localization.

### The role of Miy1 in regulating protein ubiquitination status at the plasma membrane

**Figure 2:**
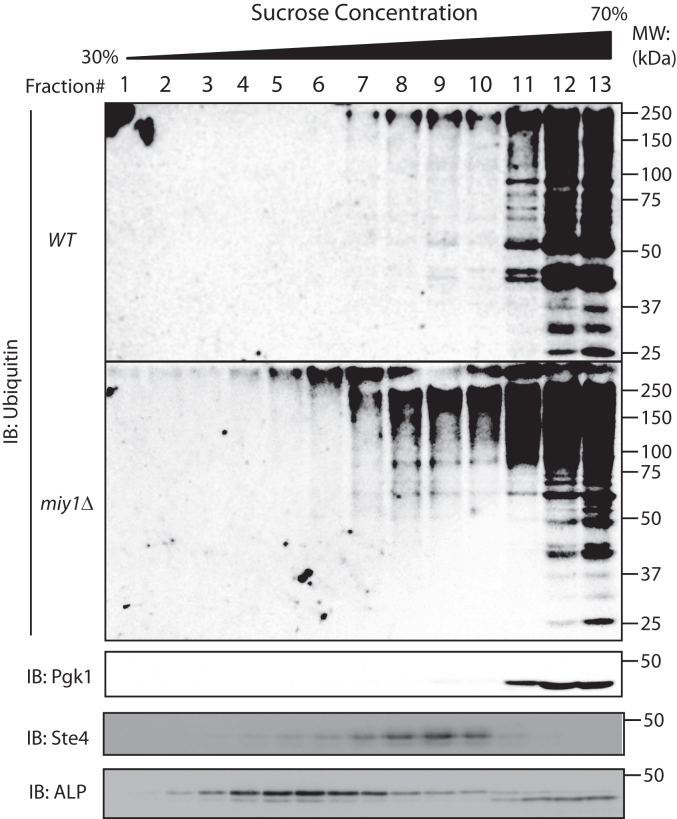
**Miy1 limits the level of protein ubiquitination in the plasma membrane.** Whole-cell extracts from either WT cells or the *miy1Δ* mutant cells grown to mid-log phase were fractionated *via* ultracentrifugation on a 30% to 70% sucrose gradient. The fractions were separated on 10% SDS-PAGE gel and analyzed by Western blotting using anti-ubiquitin (for samples from both WT and the *miy1Δ* cells), or anti-Pgk1, anti-Ste4, and anti-ALP (for samples from WT cells). Pgk1, phospho-glycerate kinase 1.

Previous research has demonstrated that recombinant Miy1 but not Miy3 possess *in vitro* activity toward K48-linked ubiquitin chain (32). Thus, to study the biological function of MINDY in yeast, initially we focused our efforts on Miy1, despite a higher expression level of Miy3. Having determined that a portion of Miy1 is plasma membrane-associated, we decided to investigate whether Miy1 participates in the regulation of plasma membrane protein ubiquitination. To this end, we prepared whole-cell extracts from WT and the *miy1Δ* mutant, fractionated them using sucrose gradient, and examined the level of protein ubiquitination of different fractions. The fractions corresponding to plasma membrane, cytosol, and intramembrane were identified using antibodies specific to a heterotrimeric G protein (Ste4), phospho-glycerate kinase 1, and the vacuolar alkaline phosphatase, respectively. We then probed the membranes with anti-ubiquitin antibody and compared the relative band intensity in the plasma membrane fractions *versus* the cytoplasmic fractions. As shown in Figure 2, in both WT and the *miy1Δ* mutants, cytosolic fractions have the highest level of ubiquitinated proteins. While the intensities of these bands are comparable between WT and the *miy1Δ* mutants, for the plasma membrane fractions and to some extent the intramembrane fractions, signals corresponding to high molecular weight ubiquitin conjugates are clearly stronger in the *miy1Δ* mutant cells. This result suggests that Miy1 limits the extent of polyubiquitination of membrane-associated proteins and potentially serves as a safeguard to protect some membrane-associated proteins from unneeded ubiquitination and degradation.

### The role of Miy1 in regulating Ste2 and Gpa1

**Figure 3:**
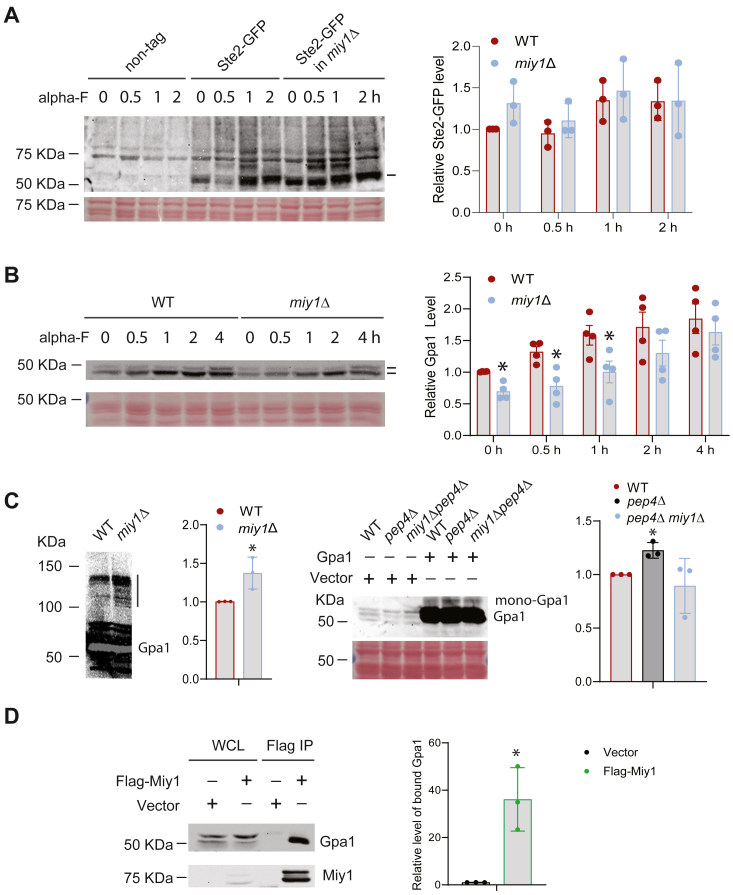
**The effect of disrupting *MIY1* on Ste2 and Gpa1.***A*, cells with or without GFP-tagged Ste2 were grown to mid-log phase and treated with pheromone for the indicated time. Whole cell extracts were analyzed by Western blot using anti-GFP to reveal GFP-Ste2 (marked as a *bar*). Equal loading was confirmed by Ponceau S staining. Quantitation from three independent experiments showing the relative level of GFP-Ste2 was shown on the *right*. The level of GFP-Ste2 in WT at time 0 was set as 1. *B*, WT cells or the *miy1Δ* cells were grown to mid-log phase, treated with pheromone for the indicated time. Whole-cell extracts were analyzed by Western blot using anti-Gpa1 to reveal Gpa1 (marked as the *bars*). Equal loading was confirmed with Ponceau S staining. Quantitation from four independent experiments showing the relative level of Gpa1 was shown on the *right*. The level of Gpa1 in WT at time 0 was set as 1. At each time point, the difference between WT and the *miy1Δ* mutants was statistically analyzed using *t* test (∗*p* < 0.050). *C*, *left panel:* WT cells or the *miy1Δ* cells were transformed with a plasmid that expresses Gpa1. Cells were grown to mid-log phase, and whole-cell extracts were analyzed by Western blot using anti-Gpa1. Polyubiquitinated Gpa1 is indicated with a *bar*. *Right panel:* cells were transformed with either empty vector or a plasmid that expresses Gpa1. Cell extracts from mid-log phase cells were analyzed by Western blotting using anti-Gpa1. Quantitation from three independent experiments showing the relative level of monoubiquitinated Gpa1 (mono-Gpa1) was shown on the *right*. The level in WT was set as 1. The difference between WT and the *miy1Δ* mutants was statistically analyzed using *t* test (∗, *p* < 0.050). *D*, WT cells were transformed with a plasmid that expresses Flag-tagged Miy1 or empty vector. Cells were grown to mid-log phase, and whole-cell lysates (WCL) were subjected to immunoprecipitation using the M2 anti-Flag resin. Both whole-cell lysates and the immunoprecipitated samples were analyzed by Western blot using anti-Gpa1 or anti-Flag (for Flag-tagged Miy1). Quantification of the level of Gpa1 in the immunoprecipitated samples from three independent experiments was shown on the *right*. The basal level of Gpa1 in vector sample was set as 1. The difference between WT and the *miy1Δ* mutants was statistically analyzed using *t* test (∗*p* < 0.050).

Ste2 is the pheromone receptor in yeast, and Gpa1 is the Gα subunit of the cognate G protein of Ste2 (39, 40). Both Ste2 and Gpa1 are among the first membrane or membrane-associated proteins known to be ubiquitinated (9, 18, 41). Ubiquitination of Ste2 is stimulated by pheromone, and it acts as a signal for triggering its endocytosis and downregulation (18). On the other hand, ubiquitination of Gpa1 occurs constitutively (42), and polyubiquitination serves to target Gpa1 protein for proteasomal degradation (19). Given the potential role of Miy1 in regulating the status of protein ubiquitination in plasma membrane, we were interested if Ste2 and Gpa1 are regulated by Miy1. We first examined a potential regulation of Ste2 by Miy1. To this end, we inserted a GFP-epitope to the C terminus of Ste2 at its genomic location in both WT and *miy1Δ* mutants. To ensure that the C-terminal tagging does not impact the function of Ste2, we compared the pheromone-induced activation of MAP kinases Fus3 and Kss1 in both tagged and nontagged strains. Adding a GFP-tag to the C terminus of Ste2 still allows pheromone-induced activation of both Fus3 and Kss1 (Fig. S1), indicating that tagging does not impact the function of Ste2. We then examined the behavior of Ste2-GFP in both WT and *miy1Δ* mutants, with and without pheromone stimulation. As shown in Figure 3*A*, pheromone stimulation does induce an accumulation of high molecular weight species of Ste2-GFP, presumably representing ubiquitinated Ste2-GFP. However, there is no clear decrease in the level of Ste2-GFP in the *miy1Δ* mutants, and the level of very high molecular weight species of Ste2-GFP representing polyubiquitinated Ste2-GFP is not increased in the mutants either. Thus, it seems that Miy1 does not have a clear role in regulating the abundance and ubiquitination of Ste2.

Next, we examined the potential effect of Miy1 in regulating Gpa1. For this purpose, we treated both WT cells and the *miy1Δ* cells with pheromone for various time, extracted proteins, and analyzed the level of endogenous Gpa1. As shown in Figure 3*B*, a clear decrease in Gpa1 was detected in the *miy1Δ* cells, which is consistent with a role of Miy1 in protecting Gpa1 protein. To examine if Miy1 regulates the ubiquitination status of Gpa1, we expressed Gpa1 in WT and the *miy1Δ* cells and monitored both ubiquitinated and nonubiquitinated Gpa1 following a previously described procedure (19, 42). As shown in Figure 3*C*, the level of polyubiquitinated Gpa1 is clearly higher in the *miy1Δ* cells. To test if Miy1 affects the level of monoubiquitinated Gpa1, we made use of the *pep4Δ* mutant, which enriches monoubiquitinated Gpa1 (19). We found that the enrichment of monoubiquitinated Gpa1 in the *pep4Δ* mutant is less prominent when the *MIY1* is also deleted (Fig. 3*C*, *right panel*). To test if Miy1 interacts with Gpa1, we expressed a Flag-tagged Miy1 in cells with Gpa1 and conducted a coimmunoprecipitation experiment. As shown in Figure 3*D*, Gpa1 clearly coimmunoprecipitates with Miy1. Together, these findings suggest Gpa1 as a target of Miy1.

### The role of Miy1 in regulating pheromone signaling

**Figure 4:**
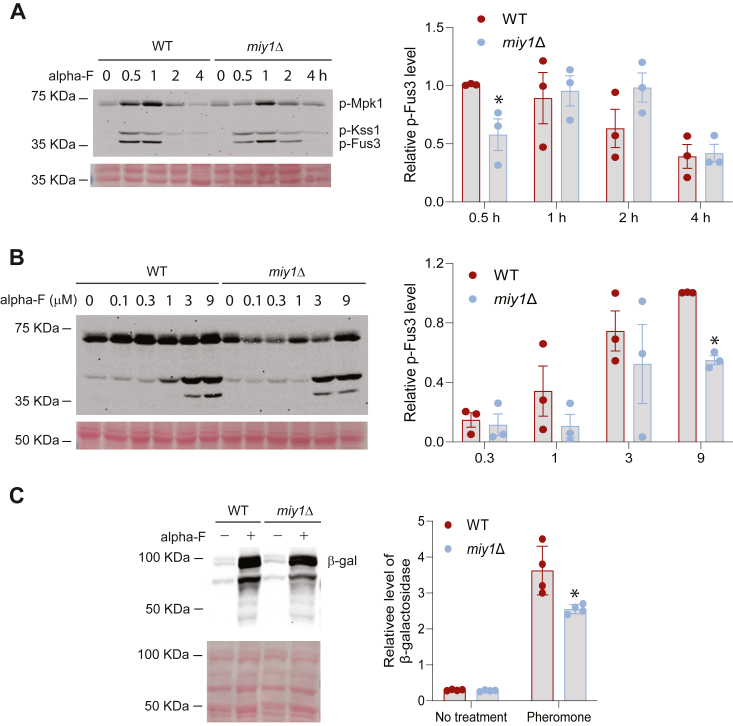
**The effect of disrupting *MIY1* on pheromone-induced MAP kinase activation.***A*, WT cells or the *miy1Δ* cells were grown to mid-log phase, treated with pheromone for the indicated time. Whole-cell extracts were analyzed by Western blot using anti-p44/p42. Equal loading was confirmed with Ponceau S staining. Quantitation from four independent experiments showing the relative level of p-Fus3 was shown on the *right*. The level of p-Fus3 in WT at time 0.5 h was set as 1. The difference between WT and the *miy1Δ* mutants was statistically analyzed using *t* test (∗*p* < 0.050). *B*, WT cells or the *miy1Δ* cells were grown to mid-log phase, treated with different concentrations of pheromone for 1 h. Whole-cell extracts were analyzed by Western blot using anti-p44/p42. Equal loading was confirmed with Ponceau S staining. Quantitation from four independent experiments showing the relative level of p-Fus3 was shown on the *right*. The difference between WT and the *miy1Δ* mutants was statistically analyzed using *t* test (∗*p* < 0.050). *C*, cells were transformed with a plasmid expressing lacZ under the control of *FUS1* promoter. Mid-log phase cells were treated or not treated with 3 μM α-factor (alpha-F) for 1 h, and cell extracts were analyzed by Western blot using anti-β-galactosidase. Quantitation from four independent experiments showing the relative level of β-galactosidase was shown on the *right*. The difference between WT and the *miy1Δ* mutants was statistically analyzed using *t* test (∗*p* < 0.050).

Having established Gpa1 as a potential target of Miy1, next we investigated if Miy1 plays any role in regulating the yeast pheromone signaling. Pheromone binding to the receptor Ste2 leads to activation of G protein composed of the Gα Gpa1 and the Gβγ Ste4-Ste18. Both Gpa1 (43) and Ste4-Ste18 (39) contribute to the full activation of a downstream MAP kinase cascade, resulting in the activation of Fus3, a mating specific MAP kinase. To examine the role of Miy1 in regulating pheromone signaling, we compared pheromone-induced activation of Fus3 in WT and the *miy1Δ* cells. The level of Fus3 activation can be conveniently examined by Western blotting using an antibody raised against the dually phosphorylated TEY motif in the activation loop of MAP kinases (44). As shown in Figure 4*A*, pheromone treatment induces Fus3 activation in both WT and the *miy1Δ* cells, however, a clear difference in the kinetics of Fus3 activation exists between WT and the *miy1Δ* cells. A delayed activation of Fus3 occurred in the *miy1Δ* cells. While the peak Fus3 activation in WT occurred 30 min post pheromone treatment, that in the *miy1Δ* cells took place 1 h post pheromone treatment. To examine if Miy1 affects the dose response, we monitored Fus3 activation in cells treated with different dosages of pheromone. As shown in Figure 4*B*, the maximal response of Fus3 activation is substantially lower in the *miy1Δ* cells.

Pheromone treatment also induces transcription of genes required for mating (39). Thus, another commonly used assay for pheromone signaling is the reporter transcription assay, in which a reporter gene *LacZ* is under the control of a pheromone-inducible *FUS1* promoter (44). To examine if Miy1 is required for pheromone-induced gene transcription, we analyzed the *FUS1*-*LacZ* reporter activity in WT and the *miy1Δ* cells. As shown in Figure 4*C*, a lower level of *FUS1-LacZ* activity is observed in the *miy1Δ* cells, indicating that a full activation of pheromone-induced gene transcription requires Miy1.

**Figure 5:**
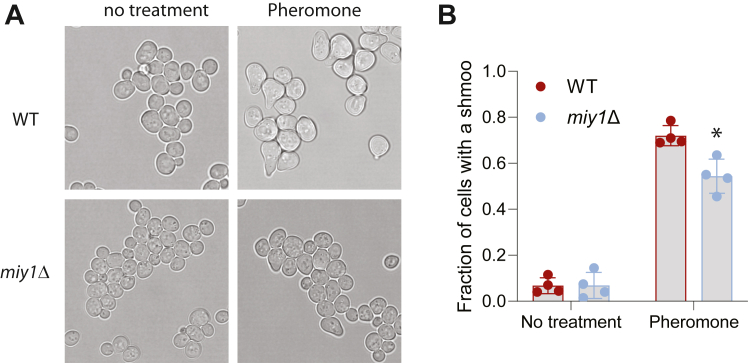
**The effect of disrupting MIY1 on pheromone-induced morphological change.** WT cells or the *miy1Δ* cells were grown to mid-log phase, treated with pheromone for 2 h. The images of the cells were taken under microscope (*panel A*), and the number of cells with a clear shmoo was counted. The data from three independent experiments were shown in *panel B*. The difference between WT and the *miy1Δ* mutants was statistically analyzed using *t* test (∗*p* < 0.050).

Prolonged pheromone stimulation in yeast leads to morphological changes, forming a pear-shaped shmoo (45). Recent work indicates that Gpa1 ubiquitination plays a critical role in shmoo formation (46), prompting us to investigate if disrupting *MIY1* has any impact on pheromone-induced shmoo formation. To this end, we grew both WT and the *miy1Δ* cells to mid-log phase, treated with pheromone for 2 h, and examined the appearance of shmoo in each cell. As shown in Figure 5, in WT cells, about 75% of cells have shmoo, whereas in the *miy1Δ* mutant cells, only about 50% of cells have shmoo.

**Figure 6:**
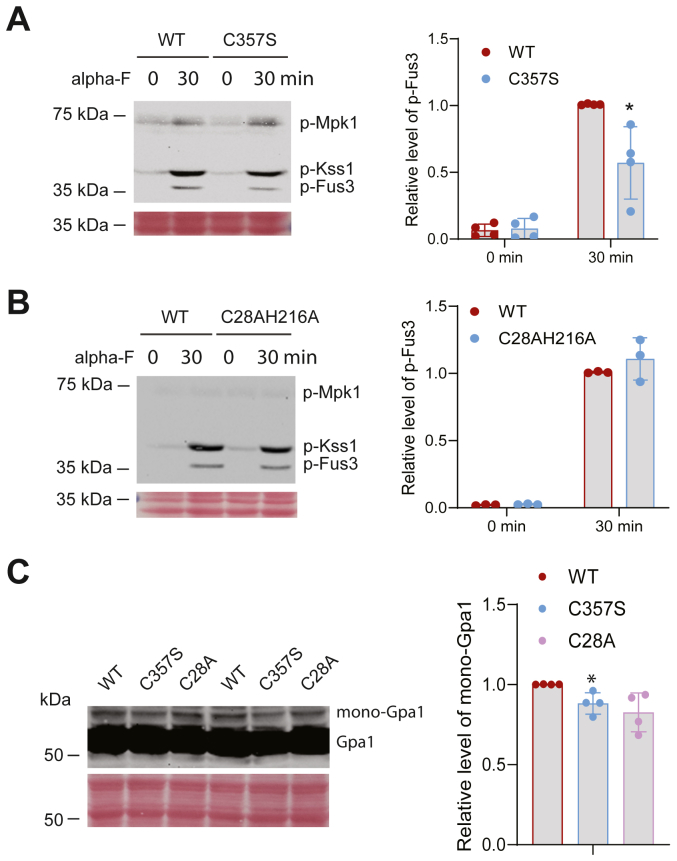
**The effect of Miy1 prenylation and catalytic activity.***A*, the *miy1Δ* cells were transformed with plasmid that expresses either WT Miy1 or its prenylation site mutant (C357S), grown to mid-log phase, treated with pheromone for 30 min. Whole-cell extracts were analyzed by Western blot using anti-p44/p42. Equal loading was confirmed with Ponceau S staining. Quantitation from four independent experiments showing the relative level of p-Fus3 was shown on the *right*. The difference between WT and the C357S mutant was statistically analyzed using *t* test (∗*p* < 0.050). *B*, the *miy1Δ* cells were transformed with plasmid that expresses either WT Miy1 or its catalytic site mutant (C28AH216A), grown to mid-log phase, treated with pheromone for 30 min. Whole-cell extracts were analyzed by Western blot using anti-p44/p42. Equal loading was confirmed with Ponceau S staining. Quantitation from four independent experiments showing the relative level of p-Fus3 was shown on the *right*. *C*, the *miy1Δpep4Δ* cells were transformed with plasmid that expresses Gpa1 and plasmid that expresses either WT or mutated Miy1 and grown to mid-log phase. Whole-cell extracts were analyzed by Western blotting using anti-Gpa1. Monoubiquitinated Gpa1 is labeled as mono-Gpa1. Quantitation from multiple independent experiments showing the relative level of ubiquitinated Gpa1 was shown. The difference between WT and the C357S mutant was statistically analyzed using *t* test (∗*p* < 0.050).

Finally, to determine if localization and catalytic activity of Miy1 is required for its role in regulating pheromone signaling, we mutated the prenylation site Cys357 to a serine (Miy1-C357S) and the catalytic residues Cys28 and His216 (32) to an alanine (Miy1-C28A or Miy1-C28AH216A) and examined their behaviors. As shown in Figure 6*A*, mutating the prenylation site leads to a diminished activation of Fus3, in a manner like the *miy1Δ* mutant. However, mutating the catalytic residues did not significantly alter the level of Fus3 activation (Fig. 6*B*). Consistent with their effects on Fus3 activation, prenylation site mutant (C357S) has a modest decrease in Gpa1 monoubiquitination (Fig. 6*C*). These findings suggest that proper localization of Miy1 is needed for its role in regulating Gpa1 ubiquitination and pheromone signaling.

## Discussion

Miy1 is a member of the MINDY family of deubiquitinating enzymes that have not been well characterized. In this study, we show that Miy1 is membrane-localized in a prenylation-dependent manner and plays a role in preventing the accumulation of ubiquitin conjugates in plasma membrane proteins. We further demonstrate that Miy1 interacts with Gpa1, the alpha subunit of a heterotrimeric G protein in yeast, modulates its ubiquitination and abundance, and impacts G protein–mediated pheromone responses.

The process of ubiquitination is catalyzed by a cascade of enzymes composed of ubiquitin activating enzyme (E1), ubiquitin conjugating enzyme (E2), and ubiquitin ligating enzyme or ligase (E3) (1, 2). Many substrates are modified with a K48-linked polyubiquitin chain, which targets the substrates for degradation in the 26S proteasome (47). Some proteins, especially those that associate with plasma membrane, are monoubiquitinated, which acts as a signal to regulate their intracellular trafficking (48, 49). As ubiquitin tends to form a chain, it raises an important question as how cells can maintain monoubiquitin signal for plasma membrane proteins. Recombinant Miy1 has been shown to have enzymatic activity toward K48-linked ubiquitin chain (32). Our findings that a portion of Miy1 is present in the plasma membrane and that Miy1 plays a role in limiting the accumulation of high molecular weight ubiquitin conjugates in the plasma membrane suggest that deubiquitination by Miy1 could represent a mechanism for maintaining the monoubiquitin signal for plasma membrane proteins.

Gpa1 is among the first identified substrates for ubiquitination. It was shown to be degraded by the N-end rule pathway (41), and our previous research indicated that Gpa1 is one of the plasma membrane–associated proteins that undergo both polyubiquitination and monoubiquitination (19, 42). Polyubiquitination of Gpa1 serves to target the protein for degradation in the proteasome, while monoubiquitination plays a role in regulating its subcellular localization (19). In this study, we find that disrupting *MIY1* leads to an accumulation of polyubiquitinated Gpa1 and a reduction of monoubiquitinated Gpa1, suggesting a role for Miy1 in maintaining the monoubiquitination of Gpa1. Consistent with our earlier finding that polyubiquitination targets Gpa1 for degradation, the *miy1Δ* mutants have an elevated amount of polyubiquitinated Gpa1 and a reduced level of endogenous Gpa1. The finding that Gpa1 immunoprecipitates with Miy1 suggests that the effect of Miy1 on Gpa1 is direct and Gpa1 is likely a target of Miy1.

The *miy1Δ* mutants show altered pheromone responses, including diminished activation of Fus3, reduced activity of *FUS1*-lacZ, and decreased shmoo formation. The role of Gpa1 in pheromone signaling is complex with at least two distinct roles documented. On one hand, Gpa1 serves to bind Ste4/Ste18 to terminate the Gβγ signaling (50). On the other hand, Gpa1 has a positive role in stimulating PI3 kinase, which in turn leads to enhanced activation of Fus3 through a mechanism that has not been fully elucidated (43). The positive signaling role of Gpa1 occurs in the endosome, and some ubiquitination pathway components, such as Dia2, were identified from a screen for mutants that impair the endosomal Gpa1 signaling (43). Since monoubiquitination is known to stimulate endocytosis of plasma membrane-associated proteins (48, 51), it is possible endosomal Gpa1 signaling requires its monoubiquitination. Therefore, the diminished level of signaling in the *miy1Δ* mutants could be a result of the reduced level of Gpa1 monoubiquitination. Our analysis also showed that mutating the prenylation signal (Cys357) but not the presumed catalytic residues (Cys28 and His216) of Miy1 impacts pheromone-induced Fus3 activation, suggesting that the localization but not the catalytic activity of Miy1 is needed for this regulation. One possibility is that Miy1 uses a different catalytic strategy and mutating Cys28 and His216 do not fully abolish its activity. Another possibility is that Miy1 may serve as an adapter molecule in recruiting other deubiquitinating enzymes to regulate the pheromone response. Identifying proteins that interact with Miy1, especially those that are regulated by pheromone stimulation, would help to elucidate the mechanism by which Miy1 impacts Gpa1 monoubiquitination and pheromone signaling.

There are two MINDY family members in yeast, termed Miy1 (32) and Miy3 (this study). While our study revealed a function of Miy1 in modulating the level of Gpa1 and pheromone signaling, the role of Miy3 remains unknown. In an early study, recombinant Miy3 did not show any activity toward ubiquitin chains (32), while recombinant Miy1 was able to process K48-linked ubiquitin chains. Thus, these two proteins have some fundamental differences in their functions. In our analysis of the *miy3Δ* mutants, we did not observe any effect on either Gpa1 levels or pheromone signaling (Fig. S2). Future studies should examine whether Miy3 purified from yeast, instead of bacteria, has any activity towards ubiquitin conjugates. There are precedents that some deubiquitinating enzymes act as protein complexes, requiring interacting protein(s) for full activity. One example is Ubp3, which requires its interacting protein Bre5 to have full activity toward its ubiquitinated substrates (52).

Extensive biochemical characterizations of human MINDY enzymes have been carried out, including the determination of their crystal structures and the mechanism by which they prefer K48-linked ubiquitin chains (32, 36). However, our understanding of their biological functions is still limited. One research revealed a potential role of MINDY enzymes in stem cell renewal (34), and several reports made connections between MINDY enzymes and cancer (53, 54, 55). Our finding that Miy1 acts in limiting the extent of ubiquitination of plasma membrane proteins including G alpha could help guide the study of human MINDY enzymes in identifying their molecular targets. One potential target of MINDY enzymes in humans is G protein, as several G alpha proteins including Gαi2, Gαi3, Gs, and Gβ1/2 are known to undergo ubiquitination (12, 13, 56, 57, 58), and accumulating evidence suggests a link between G protein mutation and cancer (59). In particular, like Gpa1 in yeast, both Gαi3 and Gβ1/2 undergo monoubiquitination (56, 58), and MINDY enzymes could similarly help maintain their monoubiquitination status.

In summary, we demonstrate in this work that the two MINDY family members in yeast are membrane-associated in a prenylation-dependent manner. One of them, Miy1, plays a role in regulating the ubiquitination and abundance of the G alpha of a heterotrimeric G protein, as well as its downstream signaling. As MINDY enzymes are highly conserved and several G alpha proteins in mammalian cells are known to be regulated by ubiquitination, it would be interesting to study whether the mechanisms uncovered here are applicable to humans.

## Experimental procedures

### Strains and plasmids

Standard methods for the growth, maintenance, and transformation of yeast and bacteria and for the manipulation of DNA were used throughout. The yeast *Saccharomyces cerevisiae* strains used in this study are BY4741 (*MATa leu2Δ met15Δ his3Δ ura3Δ*), BY4741-derived mutants lacking *MIY1, MIY3, or PEP4* (Research Genetics), BY4742 (*MAT*α *his3Δ1 leu2Δ0 lys2Δ0 ura3Δ0*)-derived strains expressing RFP-tagged Sac6, Anp1, Cop1, Chc1, Sec13, Snf7, Pex3, or Erg6. BY4741-derived mutant lacking both *MIY1* and *PEP4* was made by replacing *MIY1* with *HIS3* marker in the *pep4Δ*::G418 strain from Research Genetics.

The expression plasmids used in this study that have been described previously are pAD4M-Gpa1 and FUS1-lacZ (generously provided by Dr Henrik Dohlman, University of North Carolina). The plasmid pYES-FLAG-MIY1 that expresses N-terminal Flag-tagged Miy1 under the control of a *GAL1* promoter was constructed *via* amplifying *MIY1* ORF using PCR primers 5′-CGC GGA TCC ACT GAA ACA TCT CGA TAT AAA G-3′) and 5′ATA AGA ATG CGG CCG CAA CTC TTT TAC TAG A-3’. The PCR product was subcloned by digestion with BamHI and ligated to pYES vector described previously. Site-directed mutagenesis was used to generate various mutations as indicated. All constructs were confirmed by sequencing.

The pRS316-GFP-FLAG-MIY1 plasmid that expresses both GFP- and Flag-tagged Miy1 under its own promoter was constructed by synthesizing the gene (Gene Universal) and ligating it to pRS316 *via* BamHI and SalI sites. The GFP and Flag tags were inserted to the N terminus of the protein. The pRS316-GFP-FLAG-MIY3 plasmid that expresses both GFP- and Flag-tagged Miy3 was similarly constructed by ligating the synthesized gene to pRS316 *via* SmaI and SalI sites. The pYES-GFP-FLAG-MIY1 plasmid that expresses both GFP- and Flag-tagged Miy1 at the N terminus was constructed by the following steps. The ORF of GFP was amplified using a forward PCR primer (5′ CCC AAG CTT CCA GGA CCG ACA TTT GGG CGC 3′) and a reverse PCR primer (5′ CCC AAG CTT AGA TCG CAG TTT GTT TTT CTT AAT ATC 3′). The PCR product was subcloned by digestion with HindIII and ligation to pYES2.1/V5-His-TOPO that has been engineered to have a HindIII site. The *MIY1* fragment was amplified using a forward PCR primer (5′ GGA AGA TCG ATG GAG GAA CAA CGTGAA ATA C 3′) and a reverse PCR primer (5′ T CCC CCC GGG TCA CAA CTC GCC GAA TTC ATC 3′). The PCR product was subcloned to the above vector that has been engineered to have a BamHI site.

### Immunoprecipitation

Interaction between Miy1 and Gpa1 was examined by immunoprecipitation of FLAG-tagged Miy1 and immunoblotting with anti-Gpa1. Cells cotransformed with pAD4M-Gpa1 and pYES-FLAG-Miy1 or empty vector were grown to early-log phase, harvested by centrifugation, and resuspended in 550 μl of lysis buffer (50 mM NaPO_4_, pH 7.5, 400 mM NaCl, 0.1% Triton X-100, 10% glycerol, 0.5 mM DTT, 25 mM NaF, 25 mM glycerophosphate, 1 mM sodium orthovanadate, 10 mM *N*-ethylmaleimide, 5 mM PMSF, and one pellet of complete EDTA-free protease inhibitor mixture [Roche Applied Science] for every 50 ml of buffer). These and all subsequent manipulations were carried out at 4 °C. Cells were subjected to glass bead vortex homogenization for 30 s, repeated 10 times, and centrifuged twice at 13,000*g* for 10 min. Lysates were incubated for 2 h with a bead volume of 10 μl of anti-FLAG M2 affinity resin (Sigma) equilibrated in lysis buffer. Immunoprecipitates were collected by centrifugation at 1000*g* for 30 s, and pellets were washed with 1 ml of lysis buffer for 3 min, repeated 4 times before final resuspension in 30 μl of 2×SDS-PAGE loading buffer. Each sample was resolved by 5% SDS-PAGE and immunoblotting with anti-Gpa1 antibodies (from Dr Henrik Dohlman) at 1:1000 or anti-FLAG mAbs (from Sigma) at 1:2000.

### Sucrose gradient fractionation

Sucrose gradient fractionation was conducted as described previously (60). Spheroplasts were prepared by incubation with 20 μg/ml of zymoylase in SK buffer (1.2 M sorbitol. 0.1 M KPO4, pH 7.5) for 45 min at 30 °C. All subsequent steps were carried out at 4 °C. The resulting spheroplasts were washed once with ice cold buffer SK and resuspended in lysis buffer C (0.8 M sucrose, 20 mM triethanolamine hydrochloride [pH 7.2], 1 mM EDTA, 1 mM DTT, 0.2 mM PMSF, 1 roche protease inhibitor cocktail tablet per 50 ml), and disrupted by 15 strokes in a motorized homogenizer. The lysate was cleared of unbroken cells with 15 min of 500*g* centrifugation. Powdered sucrose was added to each sample to make 70% total sucrose, followed by mixing on a stir plate at 4 °C for 1 h. They were then carefully overlaid with 60, 50, 40, and 30% sucrose solutions, and subjected to ultracentrifugation at 190,000*g* for 19 h. Fifteen equal portions (300 μl) were drawn from the top of each tube, mixed with 2× SDS sample buffer, and boiled at 100 °C for 5 min, cooled, and resolved on 10% SDS-PAGE. Blots were probed with anti-ubiquitin or anti-K48 chain for examining the level of polyubiquitinated conjugates in different membrane fractions. Membrane fraction markers were used to establish which fractions corresponded to which membranes, anti-Ste4 (from Duane Jenness, University of Massachusetts) for plasma membrane, and anti-phospho-glycerate kinase 1 (from Jeremy Thorner, University of California) for cytosolic fractions.

### Microscopy analysis

Cells expressing GFP-tagged Miy1 or Miy3 were grown to mid-log phase. Cells were concentrated, and 10 μl of concentrated cell suspension was placed on a slide with a thin layer of 0.5% agar and visualized by fluorescence microscopy using an Olympus FV1000 laser-scanning confocal microscope.

### Western blot analysis

Gpa1 ubiquitination was monitored by transforming cells with pAD4M-Gpa1 and examining the whole-cell extracts with anti-Gpa1 antibody. For all the immunoblotting analysis, mid-log phase cells were grown on appropriate medium. Proteins were extracted *via* trichloroacetic precipitation, following procedures described previously (43). Whole-cell extracts were resuspended in boiling SDS-PAGE sample buffer (62.5 mM Tris–HCl, pH 6.8, 10% glycerol, 2% SDS, 1% 2-mercaptoethanol, and 0.0005% bromphenol blue) for 5 min. Following SDS-PAGE and transfer to nitrocellulose, the membrane was probed with antibodies to β-galactosidase at 1:1000 (from Promega, Z3783), GFP at 1:5000 (from Abcam, ab13970), Gpa1 at 1:1000 (a generous gift from Dr Henrik Dohlman, University of North Carolina), or phosphor-p44/p42 (from Cell Signaling Technology, 9101). Immunoreactive species were visualized by enhanced chemiluminescence detection (Pierce) of horseradish peroxidase–conjugated anti-rabbit IgG (Bio-Rad), anti-Rat IgG (Abcam), or anti-Chicken IgY (Abcam). All experiments have been repeated at least three times.

### Data quantification and statistical analysis

The intensities of bands from the Western blot analysis were quantified using the Image J software (https://imagej.net/ij/) from the National Institutes of Health. Where indicated, the data from three or four biological replicates were statistically analyzed by *t* test with a *p* value less than 0.050 considered significant.

## Data availability

All data are contained within the article.

## Supporting information

This article contains supporting information.

## Conflict of interest

The authors declare that they have no conflicts of interest with the contents of this article.
