# Supplementary material for: Prenylation-dependent membrane localization of a deubiquitinating enzyme and its role in regulating G protein–mediated signaling in yeast
Source: J Biol Chem. 2025 Jan 10;301(2):108180. doi: 10.1016/j.jbc.2025.108180 (PMC11847538; doi:10.1016/j.jbc.2025.108180)
Supplement: Supplementary Figure 1 [file mmc1.pdf]

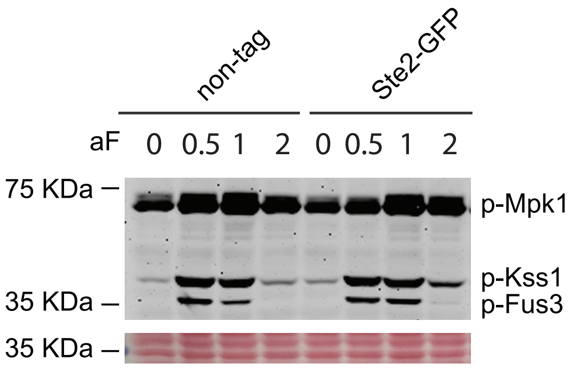

Supplementary Figure 1: cells with or without GFP-tagged Ste2 were grown to mid-log phase, and treated with pheromone α factor (αF) for the indicated time. Whole cell extracts were analyzed by western blot using anti-p44/p-42 to reveal phosphorylated Mpk1, Kss1, and Fus3. Equal loading was confirmed by Ponceau S staining.
