# Supplementary material for: Prenylation-dependent membrane localization of a deubiquitinating enzyme and its role in regulating G protein–mediated signaling in yeast
Source: J Biol Chem. 2025 Jan 10;301(2):108180. doi: 10.1016/j.jbc.2025.108180 (PMC11847538; doi:10.1016/j.jbc.2025.108180)
Supplement: Supplementary Figure 2 [file mmc2.pdf]

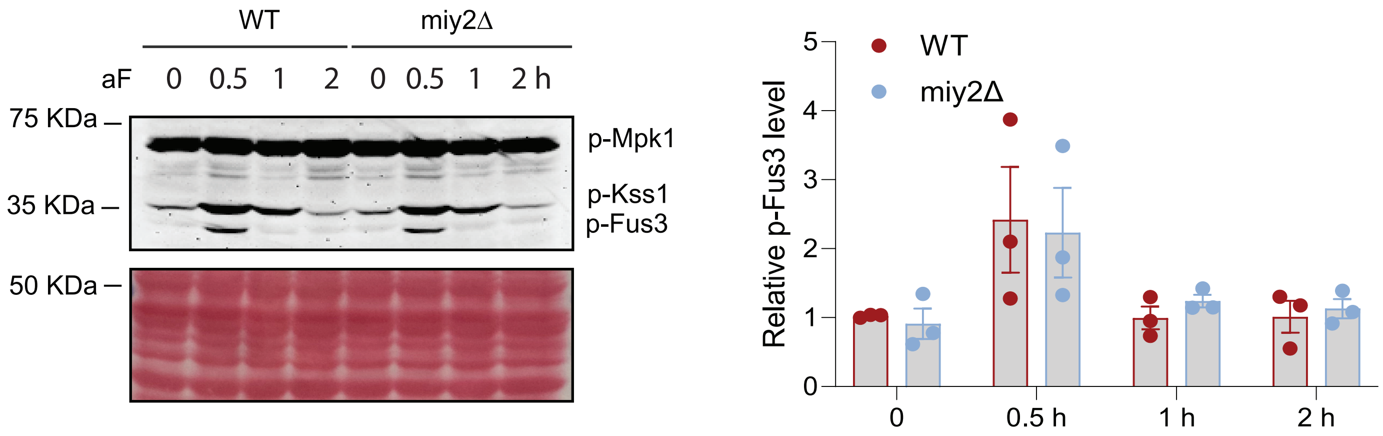

Supplementary Figure 2: cells were grown to mid-log phase, and treated with pheromone α factor (αF) for the indicated time. Whole cell extracts were analyzed by western blot using anti-p44/p-42 to reveal phosphorylated Mpk1, Kss1, and Fus3. Equal loading was confirmed by Ponceau S staining. The denisty of the band was quantified using ImageJ, and the intensity at time 0 in WT was set as 1.
